# CTRP9 knockout exaggerates lipotoxicity in cardiac myocytes and high‐fat diet‐induced cardiac hypertrophy through inhibiting the LKB1/AMPK pathway

**DOI:** 10.1111/jcmm.14982

**Published:** 2020-01-13

**Authors:** Anju Zuo, Xiaoyu Zhao, Tingting Li, Jun Li, Shengyun Lei, Jiying Chen, Dan Xu, Chengxiang Song, Tianjiao Liu, Cuigang Li, Yuan Guo

**Affiliations:** ^1^ Department of General Medicine Qilu Hospital of Shandong University Ji'nan China; ^2^ The Key Laboratory of Cardiovascular Remodeling and Function Research Chinese Ministry of Education and Chinese Ministry of Health Qilu Hospital of Shandong University Ji'nan China; ^3^ Department of Clinical Trial Research Center Jinan Central Hospital Affiliated to Shandong University Ji'nan China; ^4^ Department of Cardiology Qilu Hospital of Shandong University Ji'nan China

**Keywords:** CTRP9, lipotoxicity, LKB1/AMPK, obesity

## Abstract

CTRP9 has been reported to regulate lipid metabolism and exert cardioprotective effects, yet its role in high‐fat diet (HFD)‐induced cardiac lipotoxicity and the underlying mechanisms remain unclear. In the current study, we established HFD‐induced obesity model in wild‐type (WT) or CTRP9 knockout (CTRP9‐KO) mice and palmitate‐induced lipotoxicity model in neonatal rat cardiac myocytes (NRCMs) to investigate the effects of CTRP9 on cardiac lipotoxicity. Our results demonstrated that the HFD‐fed CTRP9‐KO mice accentuated cardiac hypertrophy, fibrosis, endoplasmic reticulum (ER) stress‐initiated apoptosis and oxidative stress compared with the HFD‐fed WT mice. In vitro, CTRP9 treatment markedly alleviated palmitate‐induced oxidative stress and ER stress‐induced apoptosis in NRCMs in a dose‐dependent manner. Phosphorylated AMPK at Thr172 was reduced, and phosphorylated mammalian target of rapamycin (mTOR) was strengthened in the heart of the HFD‐fed CTRP9‐KO mice compared with the HFD‐fed control mice. In vitro, AMPK inhibitor compound C significantly abolished the effects of CTRP9 on the inhibition of the apoptotic pathway in palmitate‐treated NRCMs. In a further mechanistic study, CTRP9 enhanced expression of phosphorylated LKB1 at Ser428 and promoted LKB1 cytoplasmic localization. Besides, silencing of LKB1 gene by lentivirus significantly prohibited activation of AMPK by CTRP9 and partially eliminated the protective effect of CTRP9 on the cardiac lipotoxicity. These results indicate that CTRP9 exerted anti‐myocardial lipotoxicity properties and inhibited cardiac hypertrophy probably through the LKB1/AMPK signalling pathway.

## 1 INTRODUCTION

Obesity is among the major risk factors for myocardial structural and functional changes, leading to high cardiovascular morbidity and mortality.^1^, ^2^ Several mechanisms that elicit obesity‐related cardiomyopathy have been proposed. Cytokines produced by the expanded adipose tissue, such as leptin and resistin, and triglyceride accumulation induce cardiac dysfunction.^3^, ^4^ Another proposed mechanism is excess supply of saturated fatty acids (FAs), such as palmitic acid. Unoxidized FAs in cardiac myocytes could produce oxidative stress and ER stress, resulting in lipotoxicty.^5^, ^6^ Oxidative stress and ER stress‐initiated apoptotic signalling pathway play crucial roles in the pathogenesis of obesity‐induced cardiac abnormality.^7^, ^8^, ^9^

AMP‐activated protein kinase (AMPK) is a major cellular sensor of energy balance in mammalian cells.^10^ Liver kinase B1 (LKB1) can act as major upstream kinases of AMPK and phosphorylate AMPK at Thr172.^11^ Under normal physiological condition, LKB1 is predominantly localized in the nucleus. Under some conditions, LKB1 is phosphorylated at Ser428 (pLKB1) and then translocated to the cytosol and subsequently induces activation of AMPK.^12^ Although several lines of evidence demonstrate that AMPK phosphorylation is attenuated in diet‐induced obesity (DIO) mouse hearts,^13^, ^14^ the mechanisms by which AMPK phosphorylation is decreased are not fully elucidated.

C1q/TNF‐related protein 9 (CTRP9) is the closest paralog of adiponectin, initially identified as an adipokine modulating metabolic and cardiovascular function.^15^ Recently, CTRP9 was reported to be abundantly produced in the heart suggesting it perhaps plays a more critical role in myocardium than APN.^16^ Several reports indicate that CTRP9 alleviates myocardial ischaemia/reperfusion (MI/R) injury, reverses post‐MI remodelling and promotes vasodilatation.^17^, ^18^, ^19^ However, CTRP9 was also shown to trigger hypertrophic cardiac remodelling and dysfunction in response to pressure overload.^20^ The discrepancy in the regulation of myocardial function by CTRP9 remains unclear. In addition, CTRP9 has been investigated to increase mitochondrial biogenesis and muscle fat oxidation suggesting it may also regulate lipid metabolism.^21^ Nevertheless, whether the protective effects of CTRP9 in HFD induced cardiac hypertrophy and lipotoxicity is unknown.

The aim of the present study was (a) to determine the functional role of CTRP9 in HFD‐induced cardiac lipotoxicity; (b) to investigate whether CTRP9 regulates myocardial hypertrophy and function in DIO mice; (c) to elucidate the underlying mechanisms responsible for the actions of CTRP9 upon cardiac lipotoxicity.

## 2 MATERIALS AND METHODS

### 2.1 Mice and diets

The experimental procedure was approved by the Institutional Ethics Committee of Shandong University and was in compliance with the Guide for the Care and Use of Laboratory Animal published by the US National Institutes of Health and Shandong University. The CTRP9‐knockout (on a C57BL/6J background) mice were generated by Shanghai Biomodel Organism Science & Technology Development Co., Ltd. WT and CTRP9‐KO mice were grown by normal chow (NC) feeding until they were 8 weeks old, after which they were randomly assigned to normal chow diet or a high‐fat (60% of total calories from fat) diet for additional 26 weeks.

### 2.2 Echocardiographic assessment

Mice were anesthetized with 1.5% isoflurane, and echocardiographic images were evaluated using the VEVO770 imaging system (VisualSonics) as described previously.^22^ Adequate depth of anaesthesia was monitored using toe reflex. The heart was imaged in the 2D mode in the parasternal long axis view with a depth of 2 cm. The left ventricular ejection fraction (LVEF), left ventricular fractional shortening (LVFS), left ventricular end‐diastolic diameter (LVEDd) and left ventricular posterior wall thickness (LVPWd) were measured by M‐type ultrasound. Heart rate was averaged over 10 cardiac cycles.

### 2.3 Cardiac histological analysis

Mouse heart tissues were harvested, washed, then fixed immediately in 4% paraformaldehyde and embedded in paraffin. Heart sections were stained with Oil red O to measure triglyceride (TG) content. Ceramides with various acyl chains (C14:0, C16:0, C18:0, C18:1, C20:0, C24:0, C24:1) were separated by HPLC with a C_18_ column (XTerra C18, 3.5 µm, 2.1 × 50 mm) and ionized in positive electrospray ionization mode as described.^23^ To evaluate the cross‐sectional area, WGA staining was examined. To detect interstitial collagen deposition, heart sections were stained with Masson's trichrome. To monitor oxidative stress status, deparaffinized sections were stained with 4‐HNE and frozen heart sections were stained with DHE. Immunofluorescence was performed for detecting the expressions of CHOP and GRP78 to assess ER stress in vivo. At least five mice per group were used for these experiments.

### 2.4 RNA isolation and real‐time PCR assays

Total RNA was extracted using Trizol (Invitrogen) according to the manufacturer's instructions. Total RNA (1 mg) was reverse‐transcribed using an oligo (dT) 16 primer to obtain cDNA. The cDNA was amplified by polymerase chain reaction (PCR). Real‐time PCR was performed with a Light Cycler 1.5 apparatus (Roche) using a Light Cycler DNA master SYBR green‐I kit according to the manufacturer's instructions. The expression of target mRNA was normalized to GAPDH.

### 2.5 Cultures of NRCMs and treatments

Primary culture of NRCMs was prepared as described previously.^24^ NRCMs were incubated in LG‐DMEM supplemented with 8% Horse Serum and 5% foetal calf serum for 24 hours after preparation and subsequently treated with a lentivirus vector containing LKB1‐siRNA at a multiplicity of infection (MOI) 80 for 48 hours. The target sequence for LKB1 siRNA was TATCTACAAGCTCTTTGAGAA, and negative control sequence was TTCTCCGAACGTGTCACGT. Thereafter, the media were replaced with fresh DMEM without lentivirus. Serum‐deprived NRCMs were pre‐incubated with recombinant gCTRP9 protein (Aviscera Bioscience) for 6 hours and then treated with palmitic acid. In some experiments, NRCMs were treated with compound C (10 µmol/L) prior to gCTRP9 protein or vehicle treatment.

### 2.6 Cell viability and cell injury evaluation

NRCMs were seeded in a 24‐well plate at a density of 1 × 10^5^ cells/well. The cell viability was determined using MTT assay (Beyotime) as previously reported. The cell damage was assessed by determining the release of LDH from the cells using a LDH Detection Kit (Beyotime) according to the manufacturer's instructions.

### 2.7 Cellular ROS production

Determination of the level of intracellular reactive oxygen species was carried out with a reactive oxygen species assay kit (Beyotime Institute of Biotechnology). NRCMs (4 × 10^5^ cells/mL) were incubated with dihydrodichlorofluorescein diacetate (DCF‐DA), delivered in serum‐free medium at 37°C for 20 minutes and then harvested in PBS. Cells treated with Rosup (50 µg/mL) for 20 minutes were used as positive controls. After loading, the fluorescence images were acquired using a fluorescence microscope (Nikon ECLIPSE TE2000‐S) in 2 minutes.

### 2.8 TUNEL staining

Apoptotic cells in NRCMs and myocardium in mice were detected by the use of a commercial DNA fragmentation detection kit (ApopTagPlus Peroxidase In Situ Apoptosis Detection Kit; Millipore) according to the manufacturer's instructions. Briefly, NRCMs were fixed with 4% paraformaldehyde for 30 minutes at room temperature. Mice heart tissue sections were deparaffinized and hydrated. The samples underwent 20 µg/mL proteinase K for 5 minutes and were washed with PBS. Then, samples were incubated with 3% H_2_O_2_ for 15 minutes. After adding the equilibration buffer, samples were incubated with TdT enzyme at 37°C for 1 hour. The samples were then incubated with antidigoxigenin conjugate at room temperature for 30 minutes. Peroxidase substrate was applied to detect apoptotic cells stained brown and normal cells appeared green (0.5% methyl green‐pyronin).

### 2.9 Preparation of cytosolic and nuclear extracts

Nuclear‐cytoplasmic fractionation was extracted using Nuclear and Cytoplasmic Protein Extraction Kit (Beyotime) according to the manufacturer's protocol. In brief, NRCMs were harvested and suspended with cytosolic proteins extraction agent A and then incubated on ice for 15 minutes followed by adding cytosolic proteins extraction agent B. After centrifugation at 4°C for 5 minutes, the supernatant was cytosolic proteins and the precipitate was suspended with nuclear protein extraction agent. After centrifugation at 4°C for 10 minutes, the supernatant was nuclear proteins.

### 2.10 Analysis of subcellular localization by microscopy

NRCMs were cultured on glass coverslips in a 24‐well plate. For LKB1 analysis, NRCMs were treated with CTRP9 (1 µg/mL) for 0, 7.5 and 15 minutes. Cells were incubated overnight at 4°C with primary antibodies for LKB1 (Santa Cruz Biotechnology) in PBS with 0.1% Triton X‐100 in a humidified chamber. Cells were washed with PBS and incubated with secondary antibody (1:200 dilution; Cell Signaling Technology) for 30 minutes at 37°C. Fluorescent images were acquired by laser scanning confocal microscopy (LSM710; Zeiss).

### 2.11 Western blot analysis

Equal amounts of protein were separated on 10% or 12% sodium dodecylsulphate‐polyacrylamide gel electrophoresis (SDS‐PAGE) and then transferred to PVDF membranes (Millipore). The membranes were blocked for 1 hours with 5% non‐fat milk at room temperature and then incubated overnight at 4°C with primary antibodies specifically against Grp78 (Abcam), CTRP9 (Aviscera Bioscience), cleaved caspase 12 (OmnimAbs), 4‐HNE (Abcam), mTOR (Abcam), p‐MTOR (Abcam), LKB1 (Cell Signaling Technology and Santa Cruz Biotechnology), pLKB1 (Ser428) (Santa Cruz Biotechnology), histone H3, cleaved caspase‐3, parp, tubulin, β‐Actin, CHOP, t‐AMPK, p‐AMPK (Thr172) all from Cell Signaling Technology. After being washed three times, the membranes were incubated with respective anti‐rabbit IgG (Abcam) and anti‐mouse IgG (Abcam) for 60 minutes at room temperature. Protein contents were visualized using an enhanced chemiluminescent reagent (Bio‐Rad).

### 2.12 Statistical analysis

Measurements are presented as means ± SEM (standard error of the mean). Differences between two groups were performed by unpaired t test, and multiple groups involved one‐way ANOVA. Differences were considered statistically significant at *P* < .05. SPSS 17.0 (SPSS) was used for statistical analysis.

## 3 RESULTS

### 3.1 Generation of CTRP9‐KO mice using the CRISPR‐Cas9 technology

**Figure 1:**
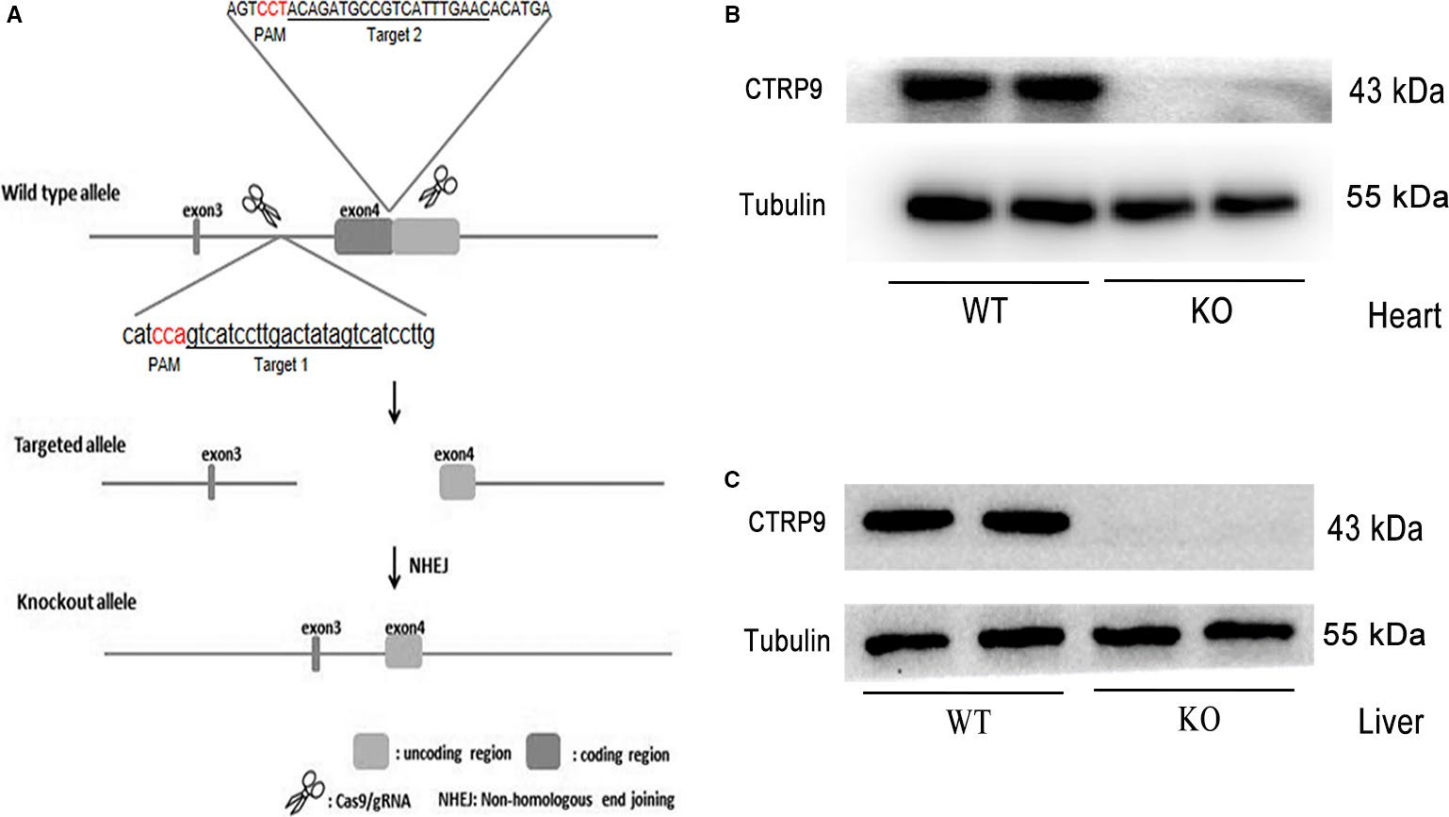
Generation of CTRP9‐KO mice using the CRISPR‐Cas9 technology. A, Schematic showing the strategy for generating CTRP9 KO mice by targeted deletion of the fourth exon of the mouse CTRP9 gene. B‐C, Western blot analysis of cardiac (B) and hepatic (C) tissues from WT and CTRP9 KO mice indicates the absence of detectable CTRP9 in KO mice. KO, knockout; WT, wild type

To generate CTRP9‐KO mice, two sgRNA (single‐guide RNA) were designed in the fourth exon of the mouse CTRP9 gene in vitro transcription (Figure 1A). Then, the microinjected zygotes with a mixture of Cas9 mRNA and sgRNA were transferred into the oviducts of some surrogate mice. Genomic DNA samples from each newborn mouse were extracted and tested for CTRP9 mutations via PCR and T‐vector. These mice that carried CTRP9 mutations were chosen to mate with C57BL/6J mice and then gave birth to heterozygous mice. Finally, we got CTRP9‐KO homozygote mice by further breeding. We examined CTRP9 expression in the cardiac and hepatic tissues of WT and CTRP9 KO mice (Figure 1B‐C).

### 3.2 CTRP9 deficiency accentuated HFD‐induced glucose and lipid metabolism disorder

**Figure 2:**
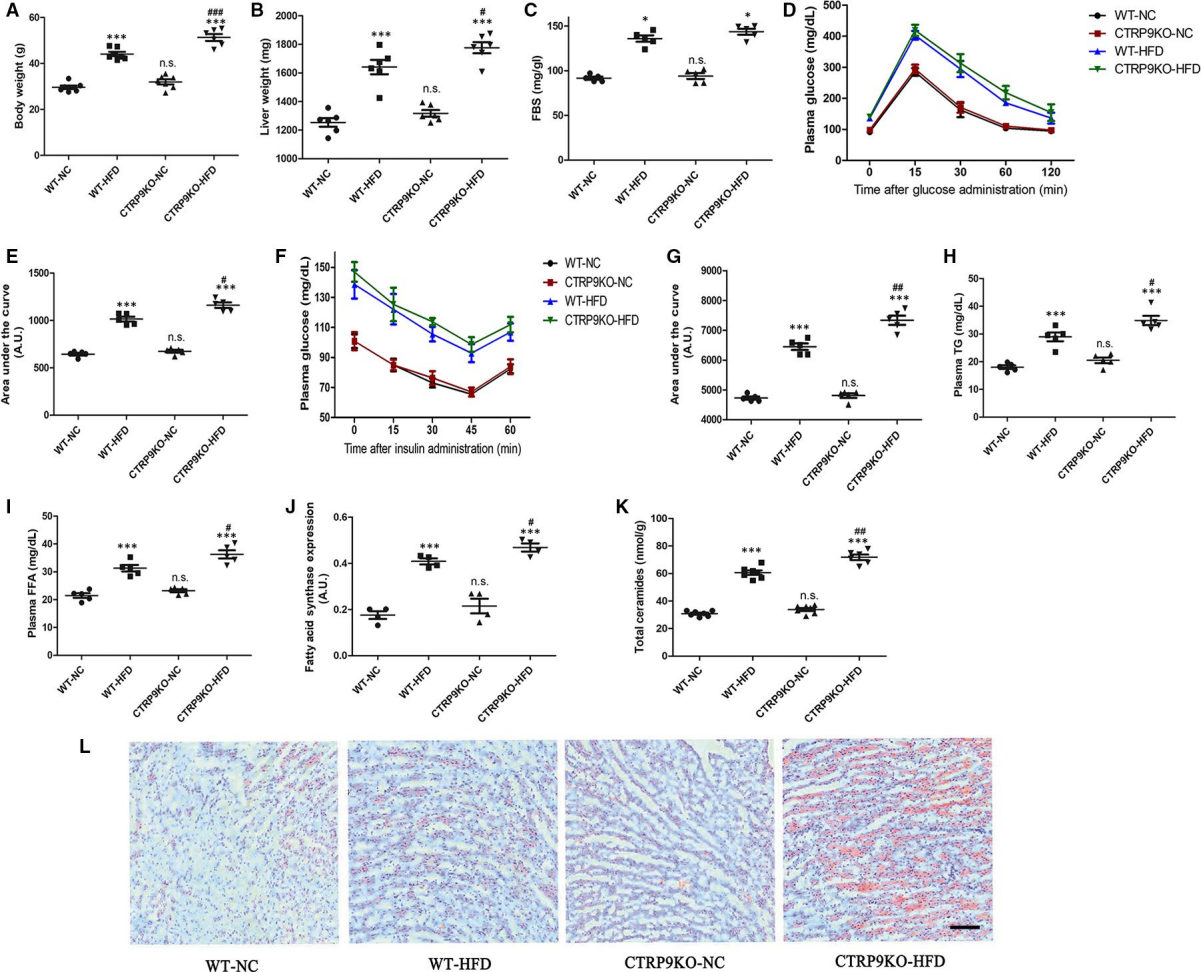
CTRP9 deficiency accentuated HFD‐induced glucose and lipid metabolism disorder. The parameters in WT and CTRP9KO mice fed normal chow (NC) or high‐fat (HF) diet for 26 wks. A, Body weight. B, Liver weights. C, fasting blood sugar (FBS) concentrations. D, intraperitoneal glucose tolerance test (IPGTT, 2 g/kg, i.p.). E, Area under the curve (AUC) of IPGTT. F, intraperitoneal insulin tolerance test (IPITT, 0.25 IU/mL, i.p.). G, Area under the curve (AUC) of IPITT. H, serum triglycerides. I, free fatty acid level. J, FAS expression. K, Quantitative analyses for myocardial contents (nmol/mg) of total ceramides. L, Representative images of Oil‐Red‐O staining. Mean ± SEM, n = 5‐8 mice per group, ^*^ *P* < .05, ^**^ *P* < .01, ^***^ *P* < .001 vs respective NC group; ^#^ *P* < .05, ^##^ *P* < .01, ^###^ *P* < .001 vs WT‐HFD group; n.s. *P* > .05 vs WT‐NC group

Circulating and cardiac CTRP9 level were decreased after 26 weeks of HFD feeding (Figure S1A‐C). High‐fat diet markedly increased body and liver weights, and the effects of which were aggravated by CTRP9 deficiency (Figure 2A and B). We observed there was little difference in fasting blood glucose in the HFD‐fed CTRP9KO mice compared with the HFD‐fed control mice (Figure 2C). Following intraperitoneal glucose and insulin challenge, serum glucose levels remained at higher levels in the HFD‐fed WT mice compared with the chow‐fed WT mice, the effect of which were augmented by CTRP9 deficiency, indicating the presence of glucose intolerance and insulin insensitivity (Figure 2D‐G). In addition, serum triglycerides (TG) and free fatty acid (FFA) were much higher in the HFD‐induced CTRP9KO mice compared with the HFD‐induced control mice (Figure 2J and K). Our data also revealed that high‐fat diet led to TG accumulation along with the up‐regulation of fatty acid synthase and total ceramides, the effects of which were exacerbated by CTRP9 deficiency (Figure 2J‐L).

### 3.3 CTRP9 knockout aggravated HFD‐induced myocardial hypertrophy and fibrosis

**Figure 3:**
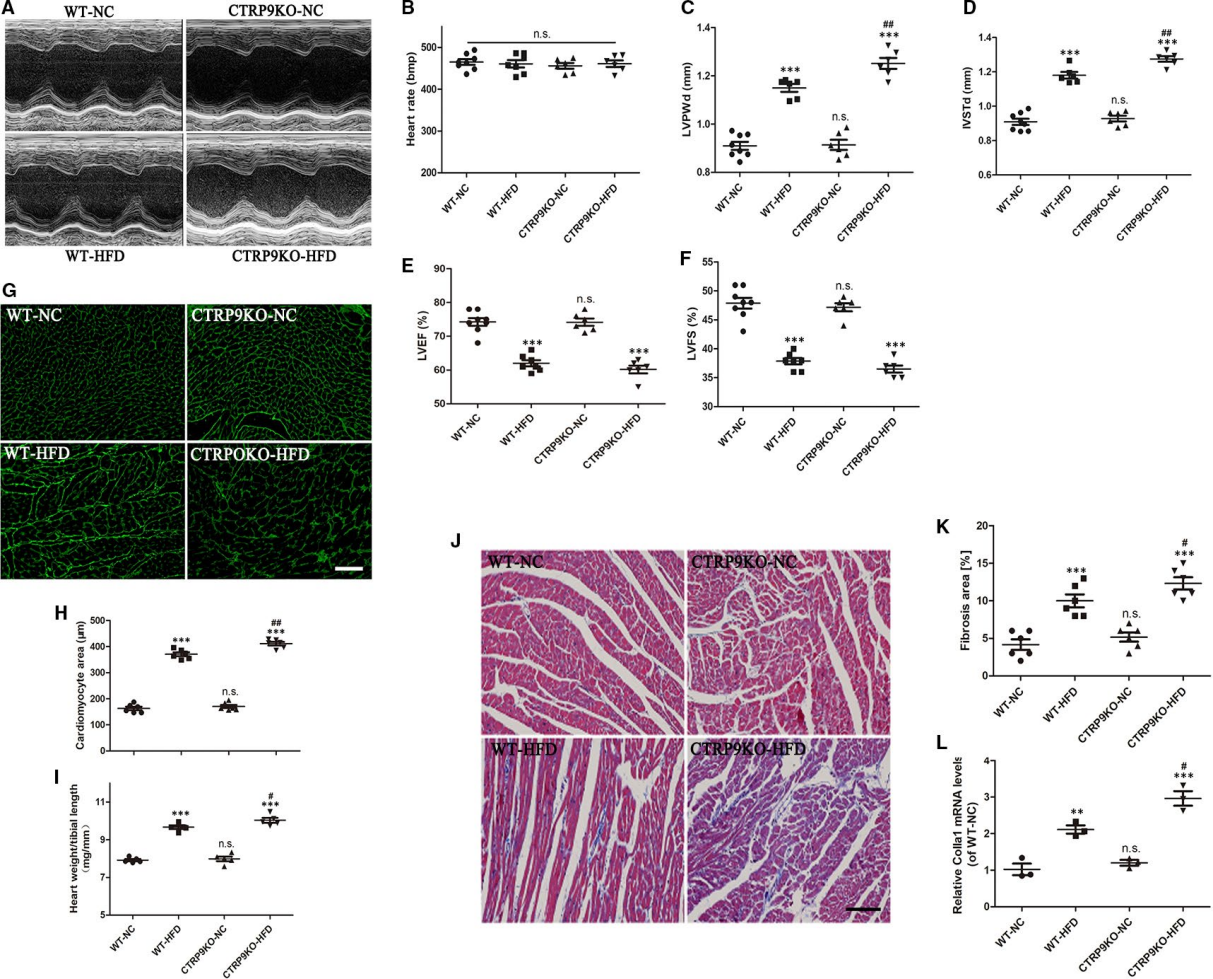
CTRP9 knockout aggravated HFD‐induced myocardial hypertrophy and fibrosis. A, Representative echocardiographic images in WT and CTRP9KO mice fed normal chow (NC) or high‐fat (HF) diet for 26 wks. B, C, D, E and F, Heart rate (B), LVPWd (C), IVSTd (D), LVEF (E) and LVFS (F), echocardiographic analysis. G and H, Representative WGA staining (40×, bar = 30 µm) of cardiac tissues and quantification of cardiomyocyte cross‐sectional area. I, The statistical analysis of the ratio of heart weight‐to‐tibial length. J and K, Representative massion staining (20×, bar = 50 µm) and quantification of cardiac fibrosis. L, colla1 expression measured by RT‐PCR. Mean ± SEM, n = 5‐8 mice per group, ^***^ *P* < .001 vs respective NC group; ^#^ *P* < .05, ^##^ *P* < .01, vs WT‐HFD group; n.s. *P* > .05 vs WT‐NC group

Echocardiographic analysis was performed after 26 weeks of normal chow or high‐fat feeding. Our experiments demonstrated HFD feeding markedly increased cardiac hypertrophy, including increases in left ventricular posterior wall thicknesses (LVPWd) and interventricular septum thicknesses (IVSTd), but did not cause cardiac systolic dysfunction (left ventricular ejection fraction [LVEF] and left ventricular shortening fraction [LVFS]) (Figure 3C‐F), which was aggravated by CTRP9 deficiency. WGA staining revealed cardiac hypertrophy was obviously enhanced in the HFD‐fed CTRP9KO mice compared with the HFD‐fed control mice (Figure 3G‐H). Consistent with the above finding, the ratio of heart weight‐to‐tibial length was further increased in the HFD‐fed CTRP9KO mice compared with the HFD‐fed WT mice (Figure 3I). Massion staining showed that HFD‐induced cardiac fibrosis was increased in the CTRP9KO than the HFD‐induced WT hearts (Figure 3J and K), which was proved by measuring Collagen Type I Alpha 1 (colla1) expression (Figure 3L). These data revealed that HFD‐induced myocardial hypertrophy and fibrosis are exacerbated in CTRP9KO mice.

### 3.4 CTRP9 knockout exacerbated ER stress‐related apoptosis in the heart of HFD‐fed mice

**Figure 4:**
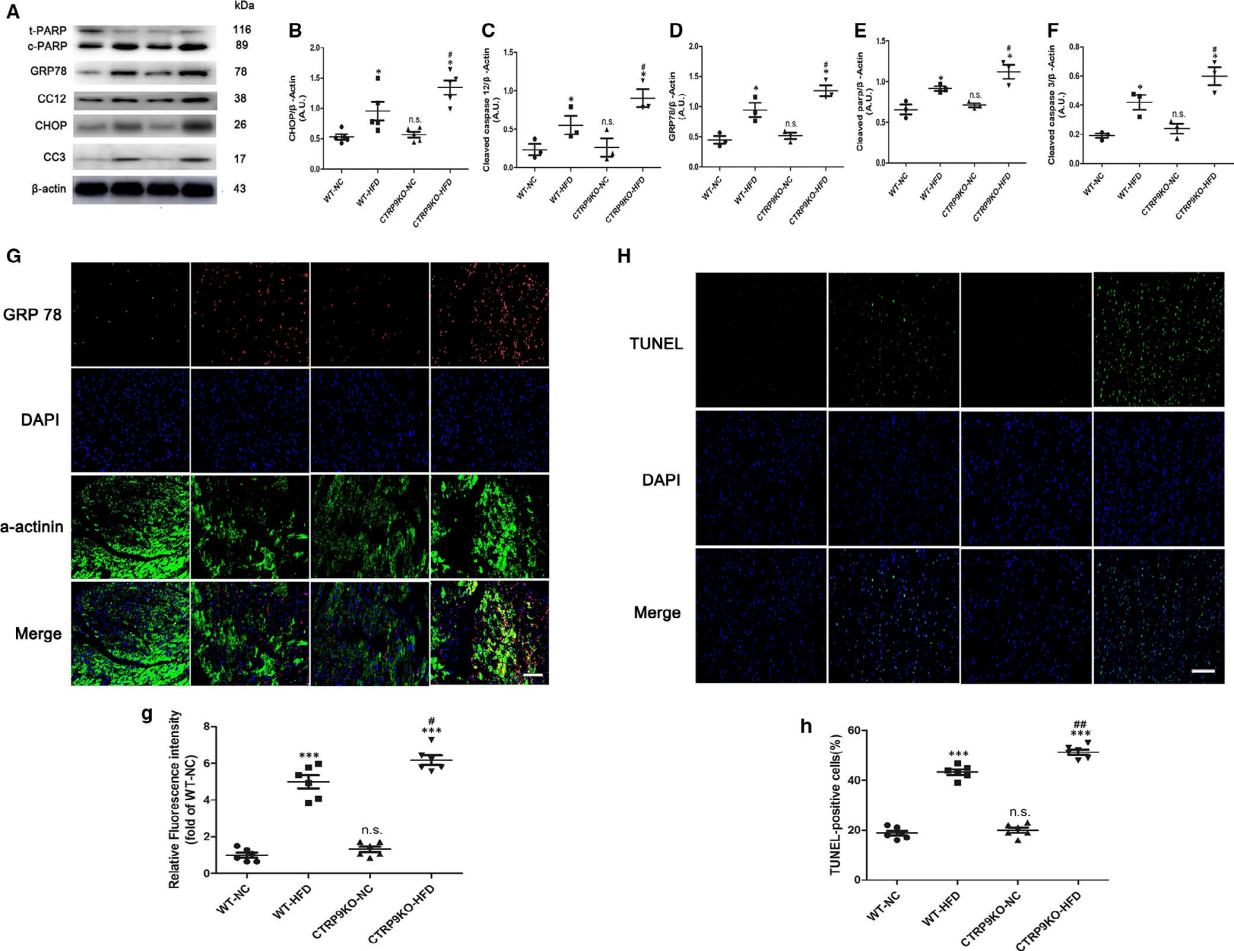
CTRP9 knockout exacerbated ER stress‐related apoptosis in the heart of HFD‐fed mice. A‐F, Lysates of heart tissue were subjected to Western blotting to analyse the expression of CHOP, GRP78, CC12 (cleaved caspase 12), CC3 (cleaved caspase 3) and c‐PARP (cleaved caspase PARP). β‐actin was used as a loading control. G and H, Representative immunofluorescence staining of GRP78 (G) (40×, bar = 40 µm). g, The relative GRP78 fluorescence intensity. H and h, Representative TUNEL staining (20×, bar = 50 µm) and related quantification of TUNEL staining. Mean ± SEM, n = 4‐6 per group, ^***^ *P* < .001 vs respective NC group; ^#^ *P* < .05, ^##^ *P* < .01, vs WT‐HFD group; n.s. *P* > .05 vs WT‐NC group. AU indicates arbitrary unit

Western blot analysis demonstrated that GRP78, CHOP, cleaved caspase 12, cleaved caspase 3 and cleaved PARP were significantly increased in the heart of HFD‐fed mice compared with the chow‐fed mice, the effects of which were augmented by CTRP9 deletion (Figure 4A‐F). Representative immunofluorescence staining of ER stress marker GRP78 in the myocardium of various groups was shown in Figure 4G. Consistent with Western blot results, quantification of GRP78 staining revealed that GRP78 was noticeably enhanced in the heart of the HFD‐fed CTRP9KO mice compared with the HFD‐fed control mice (Figure 4G). Another ER stress marker CHOP was also stained and quantified (Figure S2). Besides, the proportion of TUNEL‐positive cells was significantly increased in CTRP9KO‐HFD mice compared with WT‐HFD group (Figure 4Hand h). These data suggested CTRP9 deficiency exacerbated ER stress‐related apoptosis in HFD‐fed mouse heart.

### 3.5 CTRP9 treatment ameliorated palmitate‐induced ER stress‐related apoptosis in NRCMs

**Figure 5:**
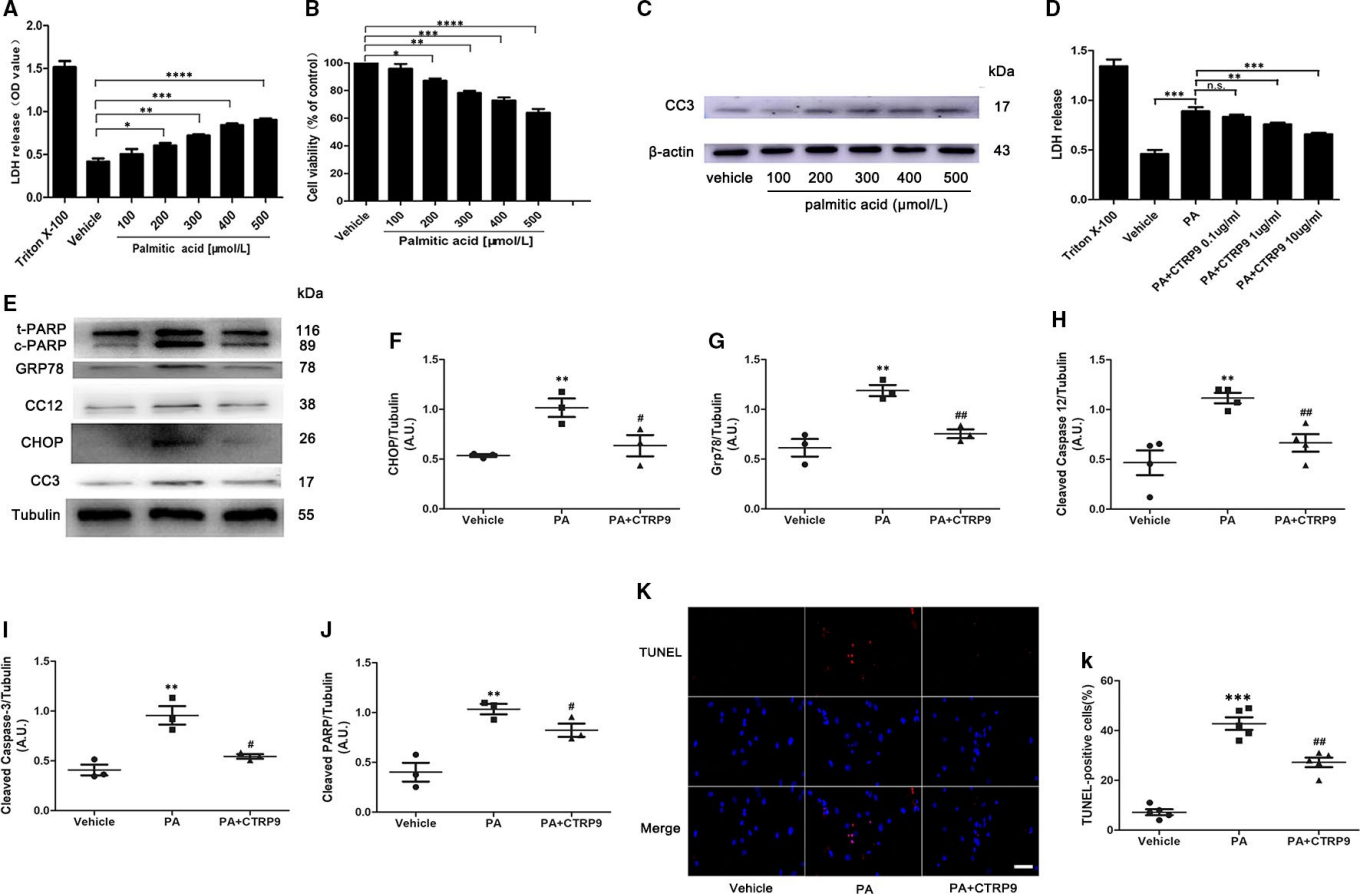
CTRP9 treatment ameliorated palmitate‐induced ER stress‐related apoptosis in NRCMs. A and B, Cell injury evaluation using lactate dehydrogenase (LDH) assays (A) and cell viability evaluation using MTT assays (B) in NRCMs. These NRCMs were stimulated with palmitate for 24 h. Mean ± SEM, n = 4 per group, ^*^ *P* < 0.05, ^**^ *P* < .01, ^***^ *P* < .001, ^****^ *P* < .0001. C, Western blotting for cleaved caspase 3 in NRCMs stimulated by various concentrations of palmitate. D, Cell injury evaluation using LDH assays in palmitate‐treated NRCMs. These NRCMs were pretreated with exogenous CTRP9 for 6 h before palmitate (400 µmol/L) stimulation. Mean ± SEM, n = 4 per group, ^*^ *P* < 0.05, ^**^ *P* < .01, ^***^ *P* < .001, ^****^ *P* < 0.0001; n.s. *P* > .05. E and a‐e, Representative Western blotting for CHOP, GRP78, cleaved caspase 12, cleaved caspase 3 and cleaved caspase PARP (E) and respective statistical analysis (a‐e). Tubulin was used as a loading control. CTRP9 1 µg/mL and palmitate 400 µmol/L were used. Mean ± SEM, n = 3‐5 per group, ^**^ *P* < .01 vs vehicle group; ^#^ *P* < .05, ^##^ *P* < .01, vs palmitate (PA) group. F and f, Representative TUNEL staining (40×, bar = 25 µm) and quantification of TUNEL staining. Mean ± SEM, n = 6 per group, ^*^ *P* < 0.05 vs vehicle group, ^#^ < 0.05 vs PA group. AU indicates arbitrary unit

Next, we stimulated NRCMs with palmitate to determine whether exogenous CTRP9 alleviated ER stress‐related apoptosis in cardiac myocytes. As shown in Figure 5A and B, palmitate stimulation resulted in elevated cardiac lipotoxicity in a dose‐dependent manner. We performed Western blotting for cleaved caspase 3 to identify the effect (Figure 5C). Exogenous CTRP9 attenuated palmitate ‐induced cardiac lipotoxicity in a dose‐dependent manner by detecting the release of LDH from the NRCMs, which was also confirmed by Western blotting for CHOP, GRP78, cleaved caspase 12, cleaved caspase 3 and cleaved PARP (Figure 5E and a‐e). In addition, TUNEL staining revealed exogenous CTRP9 significantly decreased the number of the apoptotic NRCMs. These data revealed CTRP9 ameliorated cardiac ER stress‐related apoptosis in vitro.

### 3.6 CTRP9 deficiency exacerbated oxidative stress in the heart of HFD‐fed mice

**Figure 6:**
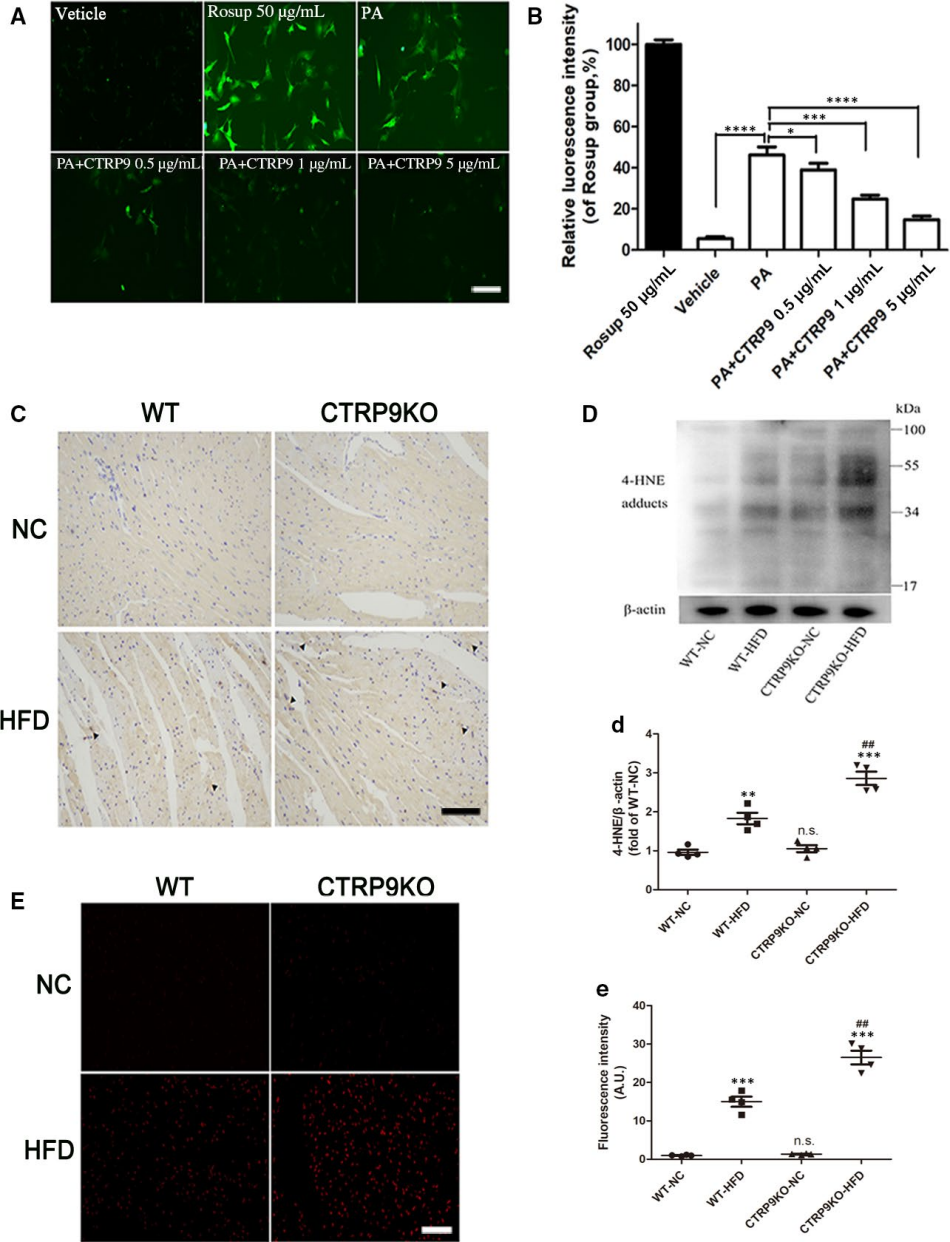
CTRP9 deficiency exacerbated oxidative stress in the heart of HFD‐fed mice. A‐B, The ROS levels in NRCMs treated with palmitate (400 µmol/L) for 12 h after pre‐incubation with exogenous CTRP9 for 6 h were evaluated by fluorescence microphototographs (10×, bar = 200 µm) and related statistical analysis for the green fluorescence intensities. CTRP9 0.5, 1 and 5 µg/mL were used in the study. Rosup was used as a ROS positive control. Mean ± SEM, n = 4‐6 per group, ^*^ < 0.05, ^**^ *P* < .01, ^***^ *P* < .001, ^***^ *P* < .0001. C, Representative immunohistochemical staining for 4‐HNE (20×, bar = 50 µm). Oxidative stress damage sites (dark brown) were marked by black triangular arrow. D and d, Representative images of Western blotting for 4‐HNE and quantifications by densitometric analysis. β‐actin was used as a loading control. E and e, Representative images of DHE staining (10×, bar = 100 µm) and fluorescence intensity analysis. Mean ± SEM, n = 4 per group, ^*^ < 0.05, ^***^ *P* < .001, vs respective NC group, n.s., not significant vs WT‐NC group. AU indicates arbitrary unit

Previous reports have shown that oxidative stress may be cell toxic for cardiac myocytes.^25^, ^26^ DCFH‐DA fluorescence probe was performed to measure ROS of different groups in vitro. As shown in Figure 6A and B, exogenous CTRP9 alleviated palmitate‐induced cardiac ROS production in a dose‐dependent manner by detecting the green fluorescence intensities of DCFH‐DA. In vivo, Western blotting and its staining for 4HNE in cardiac tissues revealed that ROS levels were higher in HFD‐fed CTRP9KO mice compared with HFD‐fed control mice (Figure 6C, D and d). We also performed dihydroethidium (DHE) staining. As shown in Figure 6E and e, DHE fluorescence intensity was significantly increased in the heart of the HFD‐fed WT mice compared with the chow‐fed WT mice, the effect of which were aggravated by CTRP9 deficiency. These data imply that CTRP9 alleviate oxidative stress in the heart of HFD‐fed mice and palmitate‐stimulated NRCMs.

### 3.7 AMPK phosphorylation is weakened in HFD‐fed CTRP9 knockout mice and palmitate‐treated NRCMs

**Figure 7:**
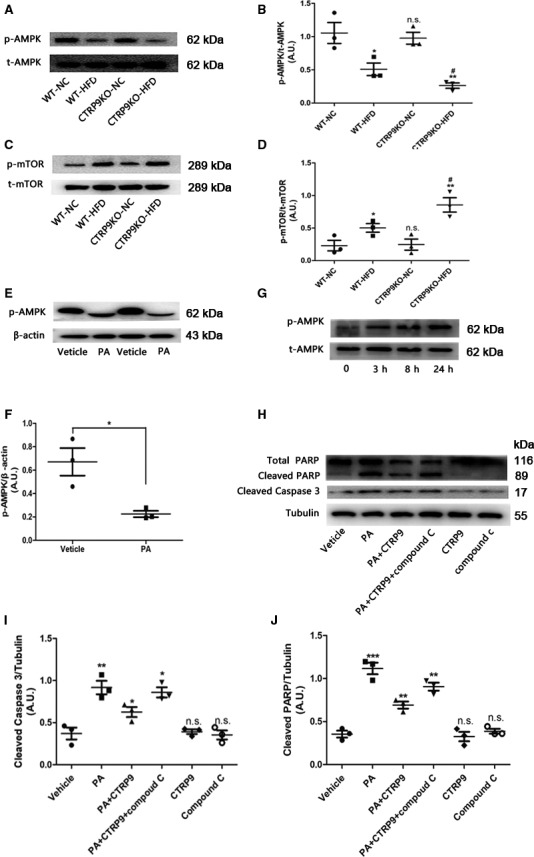
AMPK phosphorylation is weakened in HFD‐fed CTRP9 knockout mice and palmitate‐treated NRCMs. B, Representative images of Western blotting for p‐AMPK and t‐AMPK (A) and quantifications by densitometric analysis (B) of WT or CTRP9KO mice fed NC or HF diet. Mean ± SEM, n = 4 per group, ^*^ *P* < .05, ^**^ *P* < .01, vs respective NC group; ^#^ *P* < .05, vs WT‐HFD group; n.s., not significant vs WT‐NC group. C‐D, Representative images of Western blotting for p‐mTOR and t‐mTOR and related densitometric analysis. Mean ± SEM, n = 4 per group, ^*^ *P* < .05, ^**^ *P* < .01, vs respective NC group; ^#^ *P* < .05, vs WT‐HFD group; n.s., not significant vs WT‐NC group. E‐F, Representative images of Western blotting for p‐AMPK and β‐actin and the statistical analysis of the NRCMs stimulated with palmitate. β‐actin was used as a loading control. Mean ± SEM, n = 3‐5 per group, ^*^ *P* < .05, vs vehicle group. G, Representative images of Western blotting for p‐AMPK and t‐AMPK of the NRCMs treated with CTRP9 for 0, 3, 8 and 24 h (G). The concentration of CTRP9 was 1 µg/mL. H‐J, Representative images of Western blotting for cleaved caspase 3 and PARP (H) and respective statistical analysis (I and J). Tubulin was used as a loading control. CTRP9 1 µg/mL and palmitate 400 µmol/L were used. Mean ± SEM, n = 3‐5 per group, ^*^ < 0.05, ^**^ *P* < .01, ^***^ *P* < .001, vs left adjacent group, n.s., not significant vs vehicle group.AU indicates arbitrary unit

The above results showed that CTRP9 can prevent lipid metabolism disorder and HFD‐induced myocardial lipotoxicity in vivo and vitro. AMPK is a key regulator in cardiac lipid metabolism; therefore, whether CTRP9 alleviated cardiac lipotoxicity by activating AMPK was examined. AMPK phosphorylation at Thr 172 was significantly decreased in the heart of the HFD‐fed mice compared with the chow‐fed control mice (Figure 7A and B), which was consistent with the result of experiment in vitro (Figure 7E and F). In addition, AMPK phosphorylation at Thr 172 was apparently suppressed in the hearts of the HFD‐fed CTRP9KO mice compared with the HFD‐fed control mice (Figure 7A and B). Next, we assessed the phosphorylation of mTOR, a regulator of protein synthesis, which was an important repressed downstream protein of AMPK. As expected, mTOR phosphorylation was pronouncedly enhanced in the heart of the HFD‐fed CTRP9KO mice compared with the HFD‐fed control mice (Figure 7C and D). In addition, exogenous CTRP9 promoted the expression of p‐AMPK at Thr 172 in a time‐dependent manner (Figure 7G). To clarify whether the attenuation of cardiac lipotoxicity by CTRP9 resulted from AMPK activation, AMPK inhibitor compound C was used. Western blotting demonstrated compound C partially abolished the effects of CTRP9 on the inhibition of the apoptotic protein cleaved caspase 3 and PARP in palmitate‐stimulated NRCMs (Figure 7H‐J). These data suggest HFD‐induced AMPK phosphorylation is weakened in HFD‐fed CTRP9 knockout mice and palmitate‐treated NRCMs. In addition, treatment of NRCMs with CTRP9 resulted in significantly increased fatty acid oxidation through activation of AMPK, as assessed by palmitate oxidation, compared to control cells (Figure S3).

### 3.8 LKB1‐dependent AMPK activation by CTRP9 in NRCMs

**Figure 8:**
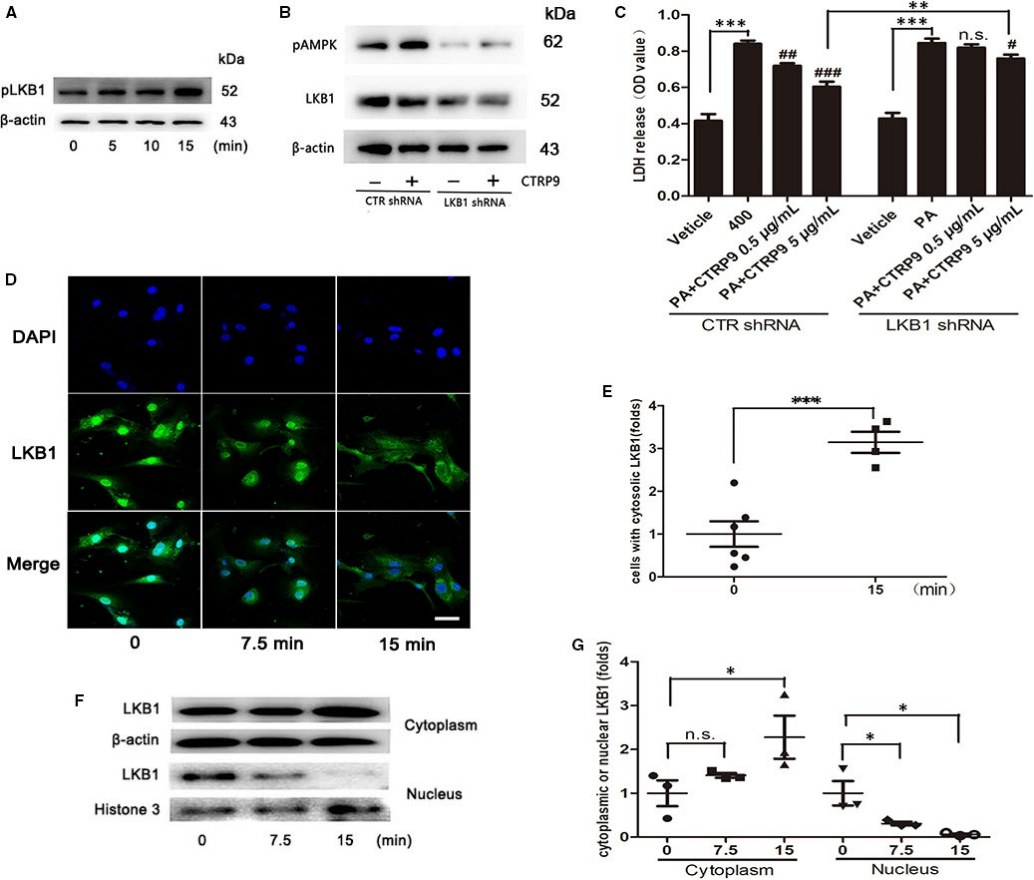
LKB1‐dependent AMPK activation by CTRP9 in NRCMs. A, LKB1 phosphorylation. Western blotting was performed on the lysates of NRCMs treated with CTRP9 for the indicated times. The concentration of CTRP9 was 1 µg/mL. B, Western blotting was performed to examine effect of LV‐LKB1‐siRNA knockdown and AMPK phosphorylation. NRCMs were treated with CTRP9 for 30 min after LV‐siRNA transfection. MOI = 80, the concentration of CTRP9 was 1 µg/mL. C, Cell injury evaluation using LDH assays in NRCMs deficient in LKB1. The NRCMs were pretreated with CTRP9 (0.5 and 5 µg/mL) for 6 h before stimulating with palmitate (400 µmol/L) for 24 h. D and E, Immunofluorescent staining of translocation of LKB1 from nucleus to the cytosols triggered by CTRP9 in NRCMs (40×,bar = 20 µm) and quantifications by densitometric analysis. The concentration of CTRP9 was 1 µg/mL. G, Representative immunoblots for LKB1 from the cytoplasm and the nucleus in NRCMs and related densitometric analysis. β‐actin and Histone‐3 were used as loading control. The concentration of CTRP9 was 1 µg/mL. Mean ± SEM, n = 3‐5 per group, ^*^ *P* < .05, ^**^ *P* < .01, ^***^ *P* < .001, ^#^<0.05, ^##^ *P* < .01, ^###^ *P* < .001, vs palmitate (PA) group. n.s., not significant

LKB1 phosphorylation at Ser428 (pLKB1) is vital to LKB1 cytoplasmic translocation from the nucleus and subsequent activation of AMPK through phosphorylation.^12^ pLKB1 was enhanced by CTRP9 treatment in a time‐dependent manner (Figure 8A), which paralleled AMPK activation. Consistently, CTRP9 did not enhance the expression of p‐AMPK in NRCMs deficient in LKB1 (Figure 8B), confirming the role of LKB1 in the activation of AMPK by CTRP9. As expected, LKB1 deficiency in NRCMs partially abolished the protective effect of CTRP9 on the palmitate‐induced lipotoxicity (Figure 8C). Immunofluorescent staining was performed to assay whether CTRP9 altered the subcellular localization of LKB1. LKB1 was observed primarily in the nucleus of NRCMs (Figure 8D and E). In CTRP9‐treated NRCMs, LKB1 was found mainly in the cytosols (Figure 8D and E). CTRP9‐promoted LKB1 nuclear export was further verified by Western blotting analysis of LKB1 in subcellular fractions (Figure 8F and G). These data indicate that LKB1‐dependent AMPK activation plays a pivotal role in the attenuation of cardiac lipotoxicity for CTRP9.

## 4 DISCUSSION

Some studies have demonstrated CTRP9 could alleviate metabolic dysfunction,^27^, ^28^ but little is known about the role of CTRP9 in HFD‐induced cardiac lipotoxicity. This study for the first time revealed that CTRP9 deficiency exaggerated lipotoxicity in cardiac myocytes and high‐fat diet‐induced cardiac hypertrophy. Besides, we demonstrated CTRP9 could exert anti‐oxidative stress and anti‐ER stress‐related apoptotic effects to alleviate lipotoxicity in NRCMs via activation of the LKB1/AMPK pathway in vitro. Therefore, we indicate CTRP9 could alleviate cardiac lipotoxicity via activation of the LKB1/AMPK pathway.

Previous studies have shown that obesity‐associated perturbations in myocardial and systemic lipid metabolism give rise to cardiac lipotoxicity.^4^, ^29^ Our data suggest that CTRP9 deficiency augmented HFD‐induced glucose intolerance, insulin insensitivity and myocardial lipid accumulation with increased body weight, serum TG and FFA, in accordance with the reported role of CTRP9 in glucose and lipid metabolism.^27^, ^30^ Clinical and experimental studies implied that obesity and type 2 DM are linked to myocardial hypertrophy.^31^, ^32^ Our data also suggest that HFD feeding results in cardiac hypertrophy. Meanwhile, our results indicate that CTRP9 knockout aggravates HFD‐triggered myocardial hypertrophy by examining echocardiography, the ratio of heart weight‐to‐tibial length and WGA staining. Nevertheless, It is opposite to a previous study that CTRP9 was shown to trigger hypertrophic cardiac remodelling during pressure overload.^20^ These results implicate that CTRP9 is in fact playing a permissive role in the complicated development of cardiac hypertrophy especially in the regulation of LKB1/AMPK pathway, which is most likely differently involved in transverse aortic constriction (TAC) vs HFD mouse models. In addition, CTRP9 replenishment is still needed to confirm this phenomenon in our study. Oxidative stress is a very vital contributor to HFD‐induced myocardial lipotoxicity. Previous studies show that excessive lipid accumulation triggered by HFD feeding could cause increased oxidative stress and subsequent apoptosis of cardiomyocytes.^1^, ^33^ Consistent with these observations, we demonstrated that the levels of ROS were higher in palmitate‐stimulated NRCMs and DIO heart. Besides, we observed exogenous CTRP9 significantly prohibited the levels of ROS in NRCMs in a dose‐dependent manner. Moreover, 4‐HNE was pronounced elevated in the heart of the HFD‐fed CTRP9KO mice compared with the HFD‐fed control mice. These findings indicate CTRP9 may exert antioxidant effect to alleviate myocardial lipotoxicity.

In addition, emerging evidence also shows ER stress has a major role in the progression of cardiac lipotoxicity.^8^, ^9^ For in vitro studies, palmitate treatment in NRCMs decreased cell viability and induced ER stress‐mediated apoptosis, which was attenuated by exogenous CTRP9 in a dose‐dependent manner. Furthermore, we demonstrated that CTRP9 deficiency exacerbated HFD‐induced ER stress‐related apoptosis by Western blotting, immunofluorescence and TUNEL staining. Taken together, CTRP9 may ameliorate cardiotoxicity partially by reducing ER stress‐related apoptosis.

Mounting evidence suggests that AMPK exerts beneficial effects such as regulation cellular lipid metabolism, anti‐oxidative stress and anti‐ER stress.^13^, ^34^ In the present study, we demonstrated that HFD feeding in vivo and palmitate treatment in vitro both reduced AMPK activity. In addition, AMPK phosphorylation was observed to be weaken in the heart of the HFD‐fed CTRP9KO mice compared with the HFD‐fed WT mice. What's more, CTRP9 was found to activate p‐AMPK in NRCMs and AMPK inhibitor compound C significantly abolished the effects of CTRP9 on the inhibition of the apoptotic pathway in palmitate‐stimulated NRCMs. These results indicate that activation of AMPK by CTRP9 may ablate myocardial lipotoxicity. In the present study, CTRP9 activated AMPK through LKB1 phosphorylation and cytoplasmic translocation, implying that LKB1 may serve as an upstream kinase of AMPK activation elicited by CTRP9. It implies that the protective effects of CTRP9 against cardiac lipotoxicity may be mediated by the LKB1/AMPK pathway.

Nevertheless, insufficiency of this study remains in that, despite CTRP9 was ablated to explore its role in cardiac lipotoxicity, our observation needs to be verified by CTRP9 replenishment in vivo. In addition, further experiments are required to figure out how CTRP9 regulates LKB1 activity.

In conclusion, our study demonstrates that CTRP9 deficiency exaggerated lipotoxicity in cardiac myocytes and high‐fat diet‐induced cardiac hypertrophy probably through inhibiting the LKB1/AMPK pathway. As CTRP9 exhibits protective effects in the heart and their levels are associated with cardiovascular events, we imply that future investigations of potential clinic applications should focus on: (a) whether the changes in CTRP9 levels can identify a population at high‐risk for cardiovascular diseases; (b) how to prevent CTRP9 levels from decreasing with ageing; and (c) how to maintain CTRP9 at appropriate levels to protect against cardiovascular diseases. More strikingly, LKB1 is recognized as a tumour suppressor gene, and inactivation of LKB1 predisposes people to various cancer.^35^ Meanwhile, a line of evidence suggests HFD‐induced obesity augments the risk of cancer as well as cardiovascular disease.^36^ Therefore, we speculate CTRP9 may have anticancer properties. Related experiments will be performed in our future studies.

## CONFLICT OF INTEREST

None.

## AUTHOR CONTRIBUTIONS

GY designed the study, ZAJ, LSY, CJY, XD and SCX performed the research and contributed to the discussion. ZAJ and ZXY wrote the manuscript. ZXY and LCG analysed the data. ZAJ wrote and edited the manuscript. LTJ and LTT and LJ reviewed and re‐edited the manuscript.

## Data Availability

Some or all dataand models used during the study are available from the corresponding author by request.
