# Supplementary figures and images for: CTRP9 knockout exaggerates lipotoxicity in cardiac myocytes and high‐fat diet‐induced cardiac hypertrophy through inhibiting the LKB1/AMPK pathway
Source: J Cell Mol Med. 2020 Jan 13;24(4):2635–47. doi: 10.1111/jcmm.14982 (PMC7028852; doi:10.1111/jcmm.14982)

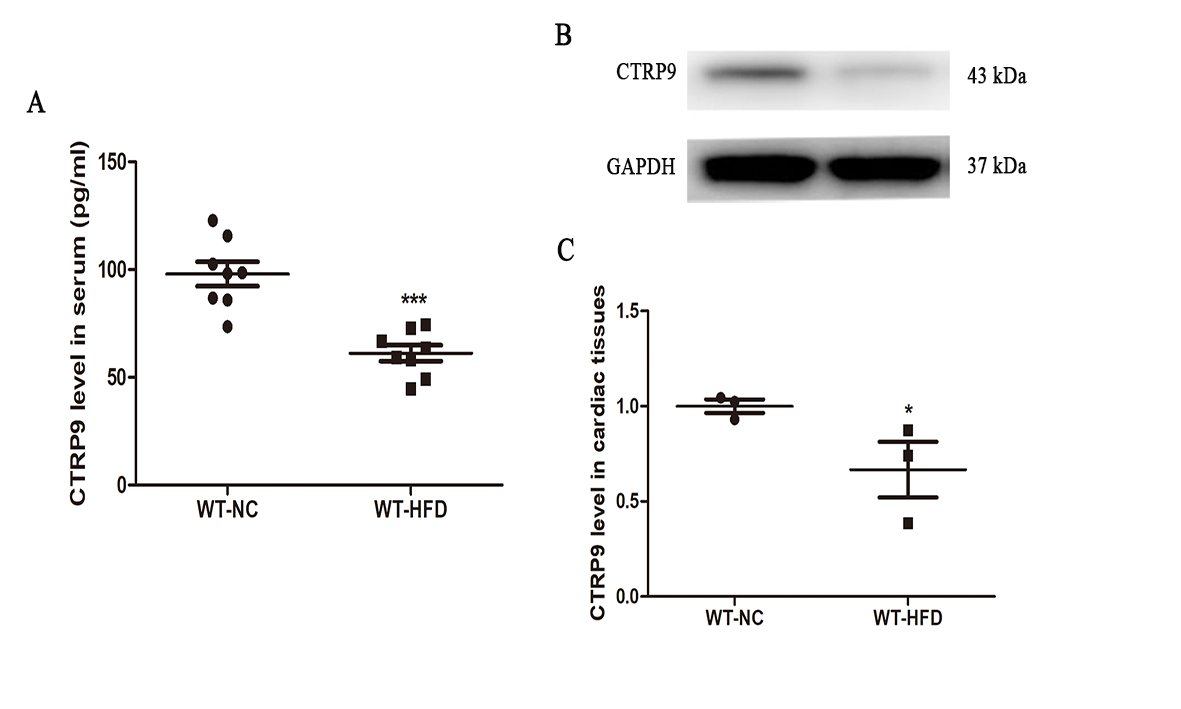

Supplement: Supplementary file 1 [file JCMM-24-2635-s001.tif]

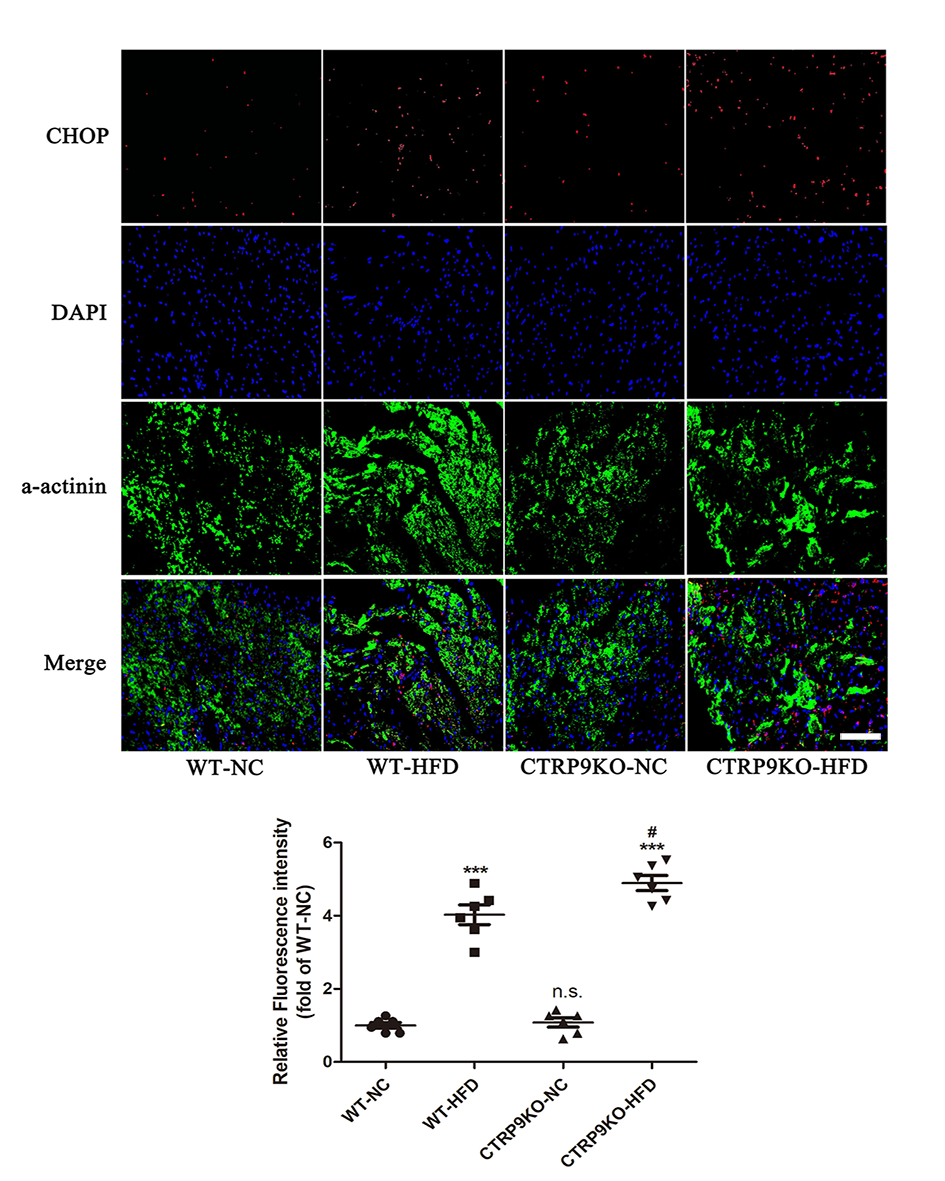

Supplement: Supplementary file 2 [file JCMM-24-2635-s002.tif]

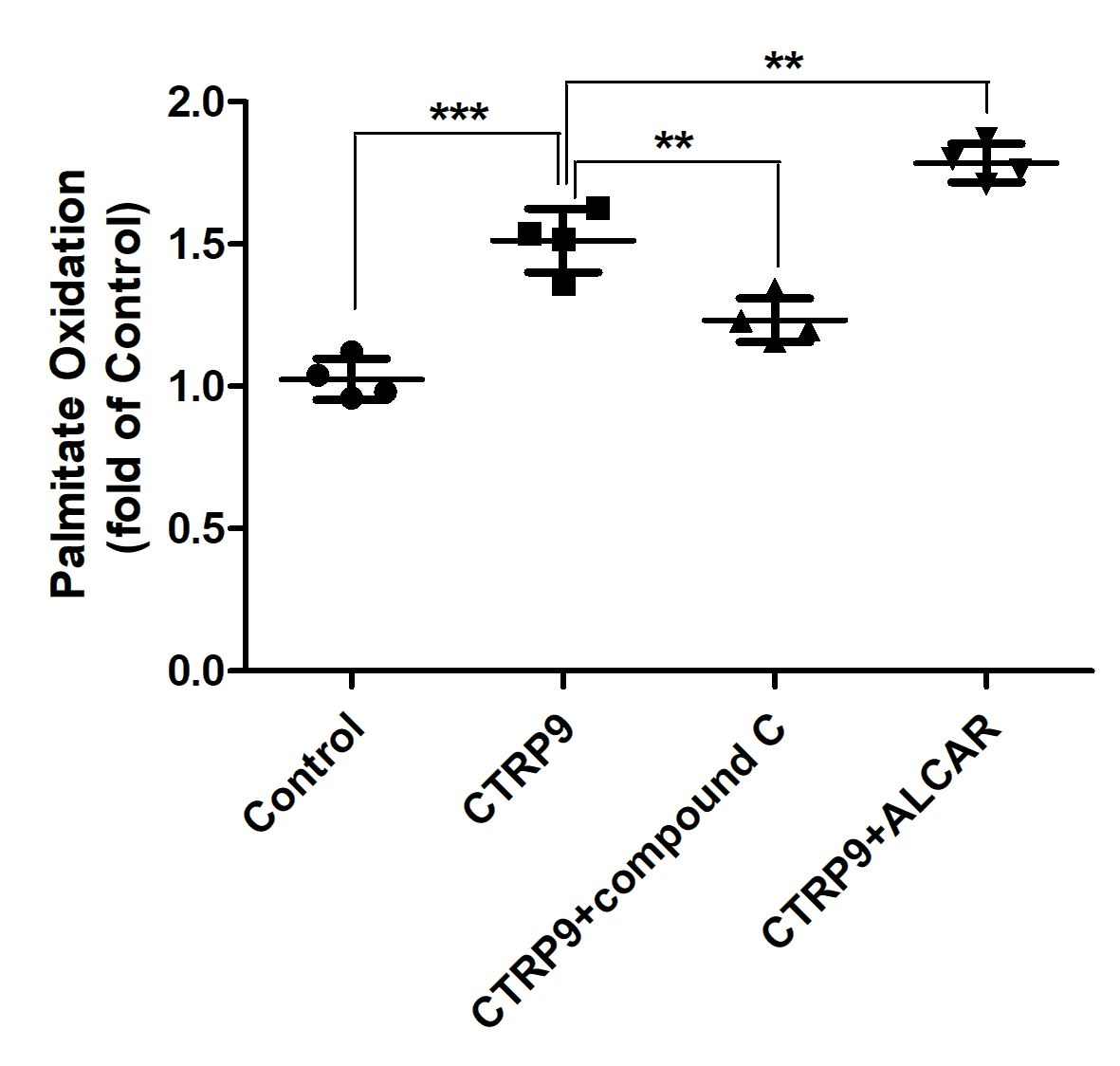

Supplement: Supplementary file 3 [file JCMM-24-2635-s003.tif]

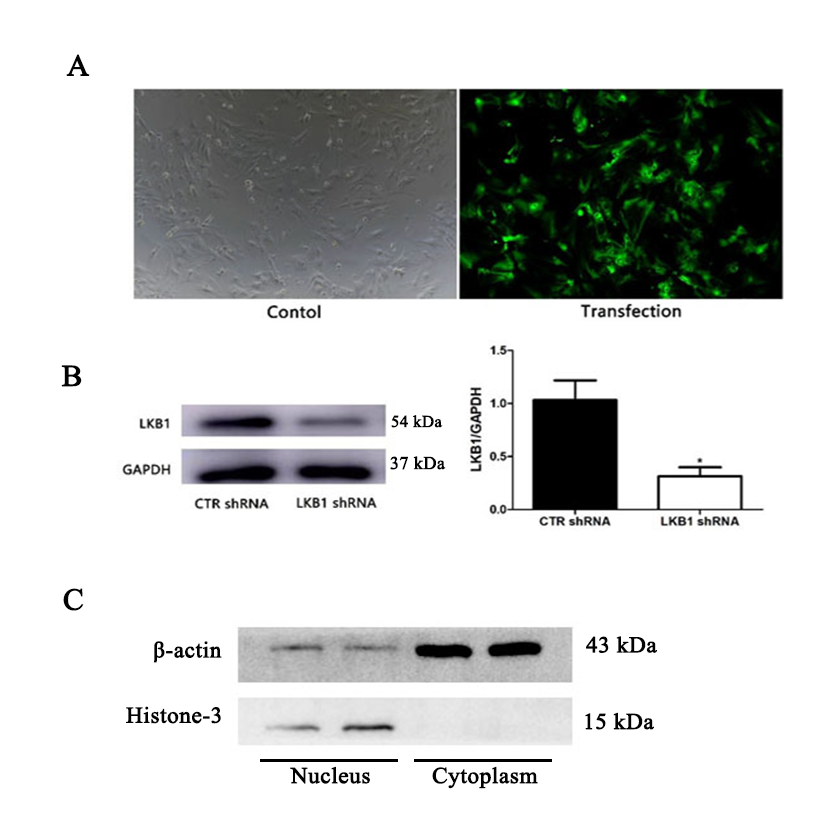

Supplement: Supplementary file 4 [file JCMM-24-2635-s004.tif]
